# Advances in schizophrenia research: glycobiology, white matter abnormalities, and their interactions

**DOI:** 10.1038/s41380-020-00961-7

**Published:** 2020-12-03

**Authors:** Julio Licinio, Ma-Li Wong

**Affiliations:** State University of New York, Upstate Medical University, Syracuse, NY 13210 USA

**Keywords:** Schizophrenia, Neuroscience

In April 2020, we published a special issue on schizophrenia, with multiple articles of considerable conceptual novelty and impact [1–19]. As we finish this 25th volume of *Molecular Psychiatry*, we have in this issue four exciting new papers that address two crucial and inter-related directions in schizophrenia research, namely glycosylation and white matter changes.

In a Perspective article, Mealer et al. point out that the role of glycosylation in schizophrenia has been emerging from genome-wide association studies (GWAS) [20]. They discuss the findings that the most robust coding variant in schizophrenia GWAS is a missense mutation in the manganese transporter gene solute carrier family 39 member 8(*SLC39A8*), which is associated with altered glycosylation patterns in humans, and variants near several genes encoding glycosylation enzymes are unambiguously associated with schizophrenia. Those include the fucosyltransferase 9 (*FUT9*), alpha-mannosidase 2 (*MAN2A1*), transmembrane O-mannosyltransferase targeting cadherins 1 (*TMTC1*), polypeptide N-acetylgalactosaminyltransferase 10 (*GALNT10*), and 3-beta-glucuronosyltransferase 1 (*B3GAT1*) genes. In this interesting perspective/hypothesis paper, the authors summarized the known biological functions, target substrates, and expression patterns of these enzymes as primers for future studies. They also highlighted a subset of schizophrenia-associated proteins critically modified by glycosylation including glutamate receptors, voltage-gated calcium channels, the dopamine D2 receptor, and complement glycoproteins. Their central hypothesis is that common genetic variants alter brain glycosylation and play a fundamental role in the development of schizophrenia.

Aberrant glycosylation in schizophrenia was also the topic of a review of 25 years of post-mortem brain studies by Williams et al. [21]. They point out the same observation noted by Mealer et al. [20] that there is recent evidence for the association of several enzymes of glycosylation with schizophrenia by GWAS, which shows the importance of glycosylation in this disease. Williams et al. then review studies conducted over the past 25 years, analyzing post-mortem brain samples, which have found evidence of aberrant glycosylation in individuals with schizophrenia. Those findings underline the role of proteins involved in both excitatory and inhibitory neurotransmission display altered glycans in the disease state, including α-amino-3-hydroxy-5-methyl-4-isoxazolepropionic acid (AMPA) and kainate receptor subunits, glutamate transporters excitatory amino acid transporter 1 and 2 (EAAT1 and EAAT2), and the γ-aminobutyric acid A (GABAA) receptor. Polysialylated NCAM (PSA-NCAM) and perineuronal nets, highly glycosylated molecules critical for axonal migration and synaptic stabilization, which are downregulated in multiple brain regions of individuals with schizophrenia. They also show that, additionally, enzymes spanning several pathways of glycan synthesis exhibit differential expression in brains of individuals with schizophrenia. These changes may be due to genetic predisposition, environmental perturbations, medication use, or a combination of these factors. Enhancing our understanding of how glycosylation is dysregulated in the brain will make it possible for us to fully ascertain its role in the development and pathophysiology of schizophrenia.

The connection between glucose, cognition, and white matter (WM) was the topic of an exciting study conducted by Zhang et al. that simultaneously examined glucose disturbances, cognitive deficits and WM abnormalities in first-episode drug-naive schizophrenia [22]. First, it is very important that they were able to conduct their study at the pre-treatment stage, thereby avoiding the confounding factor of the effects of antipsychotics on glucose metabolism. Having obtained such a critical group of drug-naïve patients, they showed that abnormal glucose metabolism, cognitive impairment and widespread disruption of WM structure occurs early in the course of schizophrenia, and is demonstrable at the onset. They conclude that interactions between glucose metabolism abnormality and WM dysconnectivity may lead to cognitive impairment. Because this study examines a sample at the onset of illness, the question then arises: how do these alterations in critical pathways evolve during the course of schizophrenia?

The WM component of that important question is answered by Cetin-Karayumak et al. in a large multicenter study involving 13 centers in North America, Europe and Asia [23]. The authors determined the pattern of age-related fractional anisotropy (FA) changes by cross-sectionally assessing the timing of the structural neuropathology associated with schizophrenia. Quadratic curves were used to model between-group FA differences across whole-brain white matter and fiber tracts at each age; fiber tracts were then clustered according to both the effect-sizes and pattern of lifespan white matter FA differences. They found that in whole-brain white matter, FA was significantly lower across the lifespan and reached peak maturation younger in patients compared to controls. Additionally, they demonstrated that three distinct patterns of neuropathology emerged when investigating white matter fiber tracts in patients: (1) developmental abnormalities in limbic fibers, (2) accelerated aging and abnormal maturation in long-range association fibers, (3) severe developmental abnormalities and accelerated aging in callosal fibers. The authors concluded by suggesting that these findings indicate that white matter in schizophrenia is affected across entire stages of the disease and that white matter alterations in schizophrenia involve dynamic interactions between neuropathological processes in a tract-specific manner.

It is remarkable that these four outstanding papers provide an in-depth understanding of both glycobiology [20, 21], white matter abnormalities [23], well as well as their interactions in schizophrenia [22]. In future issues, *Molecular Psychiatry* will continue to publish outstanding advances in schizophrenia research.

## References

[1] Koshiyama D (2020). White matter microstructural alterations across four major psychiatric disorders: mega-analysis study in 2937 individuals. Mol Psychiatry..

[2] Licinio J (2020). Advances in schizophrenia research: first special issue, 2020. Mol Psychiatry..

[3] Passos IC, Mwangi B (2020). Machine learning-guided intervention trials to predict treatment response at an individual patient level: an important second step following randomized clinical trials. Mol Psychiatry..

[4] Reay WR, Cairns MJ (2020). The role of the retinoids in schizophrenia: genomic and clinical perspectives. Mol Psychiatry..

[5] Reay WR (2020). Polygenic disruption of retinoid signalling in schizophrenia and a severe cognitive deficit subtype. Mol Psychiatry..

[6] Petrelli F (2020). Dysfunction of homeostatic control of dopamine by astrocytes in the developing prefrontal cortex leads to cognitive impairments. Mol Psychiatry..

[7] Wang HY (2020). mGluR5 hypofunction is integral to glutamatergic dysregulation in schizophrenia. Mol Psychiatry..

[8] Cai HQ (2020). Increased macrophages and changed brain endothelial cell gene expression in the frontal cortex of people with schizophrenia displaying inflammation. Mol Psychiatry..

[9] Scott MR, Meador-Woodruff JH (2020). Intracellular compartment-specific proteasome dysfunction in postmortem cortex in schizophrenia subjects. Mol Psychiatry..

[10] Radulescu E (2020). Identification and prioritization of gene sets associated with schizophrenia risk by co-expression network analysis in human brain. Mol Psychiatry..

[11] Bergman O, Karry R, Milhem J, Ben-Shachar D (2020). NDUFV2 pseudogene (NDUFV2P1) contributes to mitochondrial complex I deficits in schizophrenia. Mol Psychiatry..

[12] Chaumette B (2020). Missense variants in ATP1A3 and FXYD gene family are associated with childhood-onset schizophrenia. Mol Psychiatry..

[13] Ma L (2020). Schizophrenia risk variants influence multiple classes of transcripts of sorting nexin 19 (SNX19). Mol Psychiatry..

[14] Smeland OB (2020). Genome-wide analysis reveals extensive genetic overlap between schizophrenia, bipolar disorder, and intelligence. Mol Psychiatry..

[15] Warland A, Kendall KM, Rees E, Kirov G, Caseras X (2020). Schizophrenia-associated genomic copy number variants and subcortical brain volumes in the UK Biobank. Mol Psychiatry..

[16] Doucet GE, Moser DA, Luber MJ, Leibu E, Frangou S (2020). Baseline brain structural and functional predictors of clinical outcome in the early course of schizophrenia. Mol Psychiatry..

[17] Kumar J (2020). Glutathione and glutamate in schizophrenia: a 7T MRS study. Mol Psychiatry..

[18] Hadar R (2020). Prevention of schizophrenia deficits via non-invasive adolescent frontal cortex stimulation in rats. Mol Psychiatry..

[19] Cao B (2020). Treatment response prediction and individualized identification of first-episode drug-naive schizophrenia using brain functional connectivity. Mol Psychiatry..

[20] Mealer RG, et al. Glycobiology and schizophrenia: a biological hypothesis emerging from genomic research. Mol Psychiatry. 2020. 10.1038/s41380-020-0753-1. 10.1038/s41380-020-0753-1 PMC8081046 32377000

[21] Williams SE, Mealer RG, Scolnick EM, Smoller JW, Cummings RD. Aberrant glycosylation in schizophrenia: a review of 25 years of post-mortem brain studies. Mol Psychiatry. 2020. 10.1038/s41380-020-0761-1. 10.1038/s41380-020-0761-1 PMC8081047 32404945

[22] Zhang X, et al. Glucose disturbances, cognitive deficits and white matter abnormalities in first-episode drug-naive schizophrenia. Mol Psychiatry. 2019. 10.1038/s41380-019-0478-1. 10.1038/s41380-019-0478-1 31409883

[23] Cetin-Karayumak S, et al. White matter abnormalities across the lifespan of schizophrenia: a harmonized multi-site diffusion MRI study. Mol Psychiatry. 2019. 10.1038/s41380-019-0509-y. 10.1038/s41380-019-0509-y PMC7147982 31511636

